# Postnatal Epigenetic Alterations in Calves Persistently Infected with Bovine Viral Diarrhea Virus

**DOI:** 10.3390/v17050708

**Published:** 2025-05-15

**Authors:** Jessica N. Kincade, Dilyara A. Murtazina, Hanah M. Georges, Carolina L. Gonzalez-Berrios, Jeanette V. Bishop, Terry E. Engle, Marcela Henao-Tamayo, Jordan M. Eder, Erin M. McDonald, Darcy M. Deines, Brie M. Wright, Hana Van Campen, Thomas R. Hansen

**Affiliations:** 1 Department of Biomedical Sciences, Colorado State University, Fort Collins, CO 80523, USA; 2 Department of Animal Sciences, Colorado State University, Fort Collins, CO 80521, USA; terry.engle@colostate.edu; 3 Department of Microbiology, Immunology, and Pathology, Colorado State University, Fort Collins, CO 80521, USA; 4 Zoetis, Research Innovation Center, Fort Collins, CO 80523, USA

**Keywords:** bovine viral diarrhea virus, persistent infection, epigenomics

## Abstract

Bovine viral diarrhea virus (BVDV) is a globally prevalent pathogen causing severe detriment to the cattle industry. Vertical infection occurring before the development of the fetal adaptive immune response, before 125 days of gestation, results in an immunotolerant, persistently infected (PI) calf. It was hypothesized that epigenetic alterations observed in the splenic tissue of PI fetuses at gestational day 245 would persist into the postnatal period. White blood cell DNA from five PI and five control heifers at 4 months of age was subjected to reduced representation bisulfite sequencing and interpreted within the context of complete blood count and flow cytometry data herein. Analysis revealed 8367 differentially methylated sites contained within genes associated with the immune and cardiac system, as well as hematopoiesis. Differences observed in the complete blood counts of PI heifers include increased monocytes, microcytic anemia, and elevated platelets with decreased mean platelet volume. Flow cytometry revealed increased classical monocytes, B cells, and CD4^+^/CD8B^+^ and CD25^+^/CD127^−^ T cells, as well as decreased γδ^+^, CD4^+^, and CD4^−^/CD8B^−^ T cells. Investigation of the PI methylome provides a new perspective on the mechanisms of pathologies and provides potential biomarkers for the rapid identification of PI cattle.

## 1. Introduction

Bovine viral diarrhea virus (BVDV) is an economically important pathogen of the cattle industry [1]. There are two biotypes and two genotypes of BVDV: non-cytopathic (ncp), cytopathic (cp), BVDV1, and BVDV2, respectively. Infections with cp BVDV arise from mutated ncp strains in persistently infected (PI) cattle, cause cell death, and are mostly associated with fatal mucosal disease [2,3,4]. The ncp biotype of BVDV does not result in pathological changes when cultured and is profoundly more common than the cp biotype of BVDV. Instead, ncp BVDV infections are more often associated with reproductive losses incurred through decreased conception rates, abortions, stillbirths, and the production of weak, non-viable cattle infected in utero [5,6,7,8,9]. The bovine adaptive immune response develops between 125 and 150 days of gestation [5,10,11]. Infections occurring prior to 125 days of gestation can result in resorption of the fetus, mummification, stillbirth, or the birth of a live, PI calf [12,13,14]. These PI fetuses are known to mount a partial, albeit weak, immune response to BVDV infection, as evidenced by the diminished secretion of type I and II interferons (IFNs) [10,15,16,17,18]. However, PI fetuses ultimately fail to produce antibodies specific to the strain of BVDV that they are infected with during gestation. As a result, they are unable to clear the viral infection and shed viral particles for the entirety of their life. At birth, PI calves often have congenital defects of the brain, skeleton, heart, and thymus [10,11,12,19,20,21]. Moreover, PI calves experience severe immunosuppression that predisposes them to the development of secondary diseases [22]. Many PI cattle succumb to disease early in life, but some survive into adulthood and go on to expose all cattle they come into contact with [9]. Most BVDV management strategies focus on the identification and elimination of PI cattle. To differentiate PI cattle from transiently infected cattle within a herd, they must test positive for BVDV twice, with an interval of 3 weeks or greater in between the two [23]. Another component of BVDV management strategies focuses on the vaccination and prevention of BVDV infection. However, high antigenic diversity between the twenty-four BVDV1 and four BVDV2 subtypes renders vaccination only partially effective in BVDV mitigation [24] and produces varied clinical signs [25].

The developmental origin of health and disease theory indicates that environmental factors can have lasting effects on an organism and its associated phenotype [26]. Phenotypic plasticity is particularly high during gestation, making environmental factors, including nutrition, stress, and pathogenic exposure during pregnancy, particularly influential. One mode of action through which this influence is exerted occurs through chemical modifications of the organism’s epigenome, more specifically through the methylation of DNA. Methylation patterns are relatively stable and heritable to subsequent cells [27]. Within mammalian species, DNA methylation occurs on cytosines; 98% of these methylated cytosine nucleotides are immediately followed by a guanine nucleotide [28]. Regions of the genome rich in cytosines and guanines are known as CpG islands and are not often found outside of promoter regions in mammalian genomes [29,30]. Methylation of CpG islands located within the promoter region effectively silences gene transcription, while removal of methylation allows for the increased transcriptional binding of these sites [31]. Epigenetic data collected from PI fetal spleens revealed alterations in the methylation patterns of genes associated with the immune, neural, skeletal, and cardiac systems. These alterations within the methylome could result in the induction of postnatal defects [32]. Similarly, other vertical infections are known to induce both direct and indirect impacts on the epigenome. For example, the core protein of the hepatitis C virus binds to *AURKB* and reduces subsequent transcription [33]. Other infections, as in the case of BVDV, induce epigenetic alterations through the generation of inflammation [34]. As de novo DNA methylation and the chronic infection of PI cattle continue into the postnatal period, we hypothesized that these epigenetic alterations would continue to be present in PI calves to a greater degree at 4 months of age.

In this study, four-month-old PI heifers infected with an ncp BVDV1b isolate were assessed for differential methylation of DNA in comparison to control heifers. Differential analysis of the epigenome suggested that various physiological systems were impacted. Comparison of DNA methylation to complete blood counts (CBCs) and flow cytometry from the same calves infected with the same subtype of BVDV ensures the compatibility of data and the elimination of variability. The CBCs were consistent with chronic infection and altered hematopoiesis, such as elevated monocytes (which was corroborated by flow cytometry) and microcytic anemia. Flow cytometry results were correlated with differential methylation of immune system genes through abnormal percentages of T cell subpopulations. A compelling argument is proposed that postnatal defects associated with PI heifers are maintained by epigenetic mechanisms. In addition, previously undocumented alterations in white blood cell DNA from PI heifers have been discovered that have the potential to contribute to the loss of monetary value and premature death.

## 2. Materials and Methods

### 2.1. Animals and PI Identification

Eleven Angus cross PI heifers were identified at a local, cooperating ranch at approximately two months of age. Ear notches were collected from calves suspected to be infected with BVDV by the High Plains Veterinary Clinic in Limon, Colorado. Ear notch samples were tested using a BVDV antigen capture ELISA and RT-PCR at the Colorado State University (CSU) Veterinary Diagnostic Laboratory (Rocky Ford, CO, USA). Calves with samples that returned positive for BVDV were retested 1 month later to distinguish persistent infections and transient infections. Twelve age-matched Hereford-Angus heifers were generated at CSU, and seronegative status was confirmed at birth.

### 2.2. Blood Collection and Complete Blood Counts (CBCs)

Whole blood was collected from the jugular in K_2_EDTA tubes (Becton, Dickinson, and Company, Franklin Lakes, NJ, USA, cat # 07417). Blood samples utilized for analyses were collected from all control heifers at the same time. Blood samples from all PI heifers were collected at the same time, separately from the control heifers, due to housing distance. Blood samples were held on ice until transport to the Animal Reproduction and Biotechnology Laboratory (ARBL) at CSU. Hematological parameters for total white blood cells (WBCs), absolute cell counts, and percentages of neutrophils, basophils, eosinophils, monocytes, and lymphocytes were measured using an Element HT5 (Heska, Lovaland, CO, USA). Red blood cell (RBC) count, hematocrit, hemoglobin, mean corpuscular hemoglobin, mean corpuscular hemoglobin concentration, mean corpuscular volume, red cell distribution width, and platelets (including mean platelet volume) were also measured.

### 2.3. Isolation of White Blood Cells

The WBCs were isolated through density centrifugation. Blood samples were centrifuged at 1500× *g* for 15 min at 4 °C (Eppendorf 5804R). The buffy coat was aspirated, transferred to a tube containing 5 mL of ammonium–chloride–potassium lysing buffer (ACK; KD Medical, Columbia, MD, USA, cat#TGF-3015), and incubated for 5 min at room temperature. The WBCs were pelleted using centrifugation at 300× *g* for 10 min at 4 °C. After decanting the supernatant, the pellet was resuspended in 2.5 mL of ACK and incubated for 5 min at room temperature. Following the centrifugation, pelleting, and decanting of the supernatant, the WBC pellet was resuspended in 1X PBS with a pH of 7.4 for washing. The sample was centrifuged at 300× *g* for 10 min at 4 °C. The supernatant was discarded, and WBCs were resuspended in either 1 mL of PBS for DNA extraction or 1 mL of TRIzol^TM^ (Invitrogen, Carlsbad, CA, USA, cat#15596018) for RNA extraction.

### 2.4. Confirmation of PI Status by RT-PCR

Control heifers were tested to confirm BVDV seronegative status, while PI heifers were tested to determine the infecting strain of BVDV. The RNA was extracted from WBCs using TRIzol^TM^ reagent (Invitrogen, Carlsbad, CA, USA, cat#15596018) and treated with 6.8 units of RNase-free DNase I (Qiagen, Germantown, MD, USA, cat#79254) per sample. Each sample was then purified using the RNeasy MinElute Cleanup Kit (Qiagen, Germantown, MD, USA, cat#74204) according to the manufacturer’s instructions. RNA quantity and quality were determined using a nanodrop ND-1000 spectrophotometer (ThermoFisher Scientific, Waltham, MA, USA). All DNA samples had 260/280 ratios of 1.8 or greater. The cDNA was created from the extracted RNA using the Bio-Rad iScript Reverse Transcription Supermix (Bio-Rad, Hercules, CA, USA, cat#1708840). Using a thermocycler (Mastercycler, Eppendorf, Hamburg, Germany), the RNA/RT Supermix was held at 46 °C for 1 h before being heated to 94 °C for 4 min, according to the manufacturer’s instructions. The cDNA (1.5 µL) was combined with 0.5 µL of the forward and reverse primers and 22.5 µL of the Invitrogen PCR Supermix (Thermo Fisher Scientific, Walkham, MA, USA). Primers used to detect all BVDV isolates [21] as follows: forward 5′-CAT GCC CAT AGT AGG AC-3′ and reverse 5′-CCA TGT GCC ATG TAC AG-3′. Following 41 cycles of 10 s at 94 °C, 15 s of 50 °C, and 30 s at 72 °C, cDNA samples were held at 72 °C for 10 min before an infinite hold at 4 °C. Amplified samples were separated on a 2% agarose gel containing Gel Red (Biotium, Freemont, CA, USA, cat#41003) and visualized using a Molecular Imager ChemDoc XRS+ with Image Lab software, version 6.0.1 (Bio-Rad, Hercules, CA, USA). Samples from PI heifers were then sequentially tested for BVDV1a, BVDV1b, and BVDV2 according to Ridpath et al. [35].

### 2.5. Viral Neutralization Test

Whole blood was centrifuged at 500× *g* for 10 min at 4 °C (Eppendorf 5804R). The separated serum was placed into microcentrifuge tubes and stored at −20 °C for serology. Serum neutralizing antibody titers were determined in a microtiter plate format using cp BVDV2 (296c) and bovine turbinate (BT) cells as an indicator [16]. Each serum dilution was tested in duplicate and 100 TCID50/25 µL of the test virus was added to each well. The plates were incubated for 1 h at 37 °C and 5% CO_2_ before adding 1 × 10^4^ BT/well. The BTs in each well were scored for cytopathic effects after an additional 72 h of incubation. Each titration assay included duplicates of the control calf sample, a positive control serum of known titers, cell controls, and the inoculum.

### 2.6. Reduced Representation Bisulfite Sequencing (RRBS)

The DNA was isolated from WBCs using the Qiagen DNeasy Blood and Tissue Kit (Qiagen, Germantown, MD, USA) according to the manufacturer’s instructions. Extracted DNA from 5 randomly selected control heifers and 5 randomly selected PI heifers was sent to Zymo Research (Irvine, CA, USA) for genome-wide classic RRBS.

The following methods were completed by Zymo Research. Samples were digested with 30 units of MspI (NEB; Ipswich, MA, USA) and purified with DNA Clean & Concentrator-5 (Zymo Research). Fragments were ligated to pre-annealed adapters with cytosine replaced with 5′-methyl-cytosine according to Illumina’s guidelines. Ligated fragments greater than 50 base pairs were recovered with the DNA Clean & Concentrator-5 (Zymo Research), then bisulfite-treated with the EZ DNA Methylation-Lightning Kit (Zymo Research). Samples were subjected to PCR with Illumina indices, and products were then purified with DNA Clean & Concentrator-5 (Zymo Research). The size and concentration of samples were confirmed with the Agilent 2200 TapeStation (Agilent Technologies, Inc., Santa Clara, CA, USA), and libraries were sequenced on an Illumina NovaSeq 6000 (Novogene Co., Sacramento, CA, USA).

### 2.7. Methylation Bioinformatics and Pathway Analysis

DNA samples from 5 randomly selected control and PI calves were submitted to Zymo Research for reduced representation bisulfite sequencing (RRBS). Raw binary alignment and map (BAM) files were received from Zymo Research. Using the ‘methylKit’ package in R (version 4.4.0) [36], these files were aligned to the most recent bovine genome available through the University of California, Santa Cruz (ARS-UCD1.2/bosTau9). Quality control and gene ontology data and plots were generated with the ‘clusterProfiler’ and ‘org.Bt.eg.db’ packages in R [37,38]. The package methylKit selects only the CpG sites identified in all samples for comparison. Data were normalized by median coverage, filtered by a standard deviation of 2 to remove sites that did not include variation, and assessed for outliers. Clustering was performed at this stage to demonstrate differences in methylation between groups. Correlation was also performed here, with all Pearson correlation coefficients ≥ 0.95. Differentially methylated sites (DMSs), CpG islands, and promoter regions were identified using the ‘methylKit’ package in R using logistic regression. Per the ‘methylKit’ default, read coverage is weighted during differential comparison to remove variation due to sample heterogeneity. Methylation sites were identified as DMSs if they satisfied the condition of *q* < 0.01 and the methylation was varied in at least 25% of the treatment sample population (|meth.diff| > 25). CpG islands and promoter regions were identified if they satisfied the conditions of *q* < 0.01 and |meth.diff| > 15. Gene identifications were associated using the ‘genomation’ package in R [39]. Deconvolution was not necessary for this analysis, as the methylation of specific cell types was not compared. Raw files are available in the NCBI GEO Database (GSE271244). Pathway analysis for methylation data was performed using Ingenuity Pathway Analysis (IPA; Qiagen, Germantown, MD, USA).

### 2.8. Spectral Cytometry

Whole-blood samples were collected in K_2_EDTA tubes and processed by Zoetis Inc. (Kalamazoo, MI, USA). The RBCs were lysed using 1X RBC lysis buffer (eBioscience, San Diego, CA, USA, cat# 00-4300-54). Samples were centrifuged at 500× *g* for 5 min. The WBCs were resuspended in 1 mL of DPBS (ThermoFisher, Waltham, MA, USA, cat# 14190-136). Another 10 mL of DPBS was added to the suspension before centrifugation at 500× g for 5 min. The supernatant was aspirated, and the cell pellet was resuspended in 1 mL of DPBS and pipetted through a cell-strainer cap into a 5 mL round-bottom tube (Corning, Corning, NY, USA, cat# 352235). The cell suspension was diluted 1:20 into DPBS and counted using a Vi-CELL BLU (Beckman Coulter, Loveland, CO, USA). Viable cells were seeded into a 96-well V-bottom cell culture plate (Corning, Corning, NY, USA, cat# 3894) at a concentration of 1 × 10^6^ cells per well. The remaining cells were suspended in Serum Free Cell Freezing Medium (ATCC, Manassas, VA, USA, cat# 30-2600) and stored at −80 °C. The total volume of each well was brought up to 200–300 µL using DPBS prior to centrifugation of the plate. The supernatant was discarded. Cells were resuspended in 100 µL of Fixable Viability Stain 620 (BD Biosciences, Franklin Lakes, NJ, USA, cat# 564996) at a concentration of 1:1000 and allowed to incubate on ice in the dark for 10 min. Afterwards, 200 µL of FACS buffer (BD Biosciences, Franklin Lakes, NJ, USA, cat# 554657) was added to each well. Samples were centrifuged and the supernatant was discarded. Cells were resuspended in 100 µL of the staining master mix (Table 1) and incubated for 20 min on ice in the dark. Following incubation, 200 µL of FACS buffer was added, cells were centrifuged, and the supernatant was discarded. Cells were washed in 300 µL of FACS buffer and centrifuged. The supernatant was discarded before resuspension in 100 µL of diluted stabilizing fixative (1:3, BD Biosciences, Cat# 338036). After 5 min of incubation at room temperature, 100 µL of FACS buffer was added prior to centrifugation and the discarding of the supernatant. Cells were resuspended in a final 200 µL of FACS buffer and held at 4 °C in the dark until transfer to the CSU Flow Cytometry Core for analysis using a 4L 16V-14B-10YG-8R Cytek Aurora spectral cytometer (Cytek Biosciences, Fremont, CA, USA).

Data were gated using FlowJo (BD Biosciences, Franklin Lakes, NJ, USA). Flow cytometry data in the form of frequencies relative to the parent population were compared in GraphPad Prism 10 (GraphPad Software, San Diego, CA, USA).

### 2.9. Statistical Analysis

Statistical analyses of CBCs and flow cytometry values were performed in GraphPad Prism 10 (GraphPad Software, San Diego, CA, USA). Grouped values were tested for normality using the Shapiro–Wilk test. Outliers were identified using the ROUT test with a false discovery rate (q-value) of 1%. Outliers were removed prior to the calculation of means and significance. Data with normal distribution and equal variances were subjected to an unpaired, parametric T-test. Due to differing sample numbers (control *n* = 12, PI *n* = 11), all parameters were tested for equal variances. When the variance is not significantly different between the control and PI group, as determined by an F-test, a Student’s T-test was used. When variances were unequal, Welch’s correction was applied to minimize the type I error rate. In cases where the data were not normally distributed, a nonparametric Mann–Whitney test was utilized. Differences between controls and PI heifers were considered significant if *p* < 0.05. Data are presented as the mean ± standard error of the mean (SEM).

## 3. Results

### 3.1. BVDV Identification and Serology

Eleven PI heifers were confirmed to be positive for BVDV1b through RT-PCR genotyping at branding and 1 month following branding [35]. Twelve age-matched control heifers housed at CSU facilities were tested to confirm seronegative status upon birth.

### 3.2. DNA Methylation

**Figure 1:**
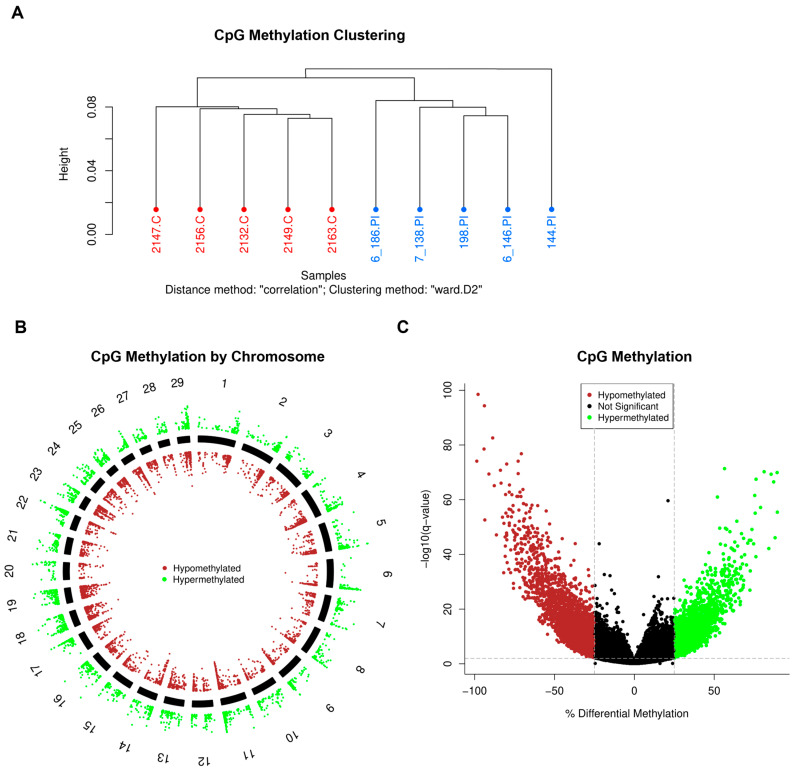
Differential methylation of CpG sites in PI calves. The CpG sites are defined as sites containing a ‘CCGG’ motif within the genome. (**A**) Clustering of samples based on percent methylation per base. Control heifers are denoted in red and heifer ID numbers are followed by ‘.C’. Persistently infected (PI) heifers are denoted in blue and animal ID numbers are followed by ‘.PI’. (**B**) Visualization of differentially methylated CpG sites identified in PI heifers compared to controls by chromosome. Each data point represents a differentially methylated CpG site with respect to its location on the chromosome. Hypermethylated CpG sites are denoted in green and are positioned outside of the solid chromosome bars. Hypomethylated CpG sites are denoted in red and are positioned within the solid chromosome bars. (**C**) Visualization of the hypermethylated and hypomethylated CpG sites identified in PI cattle compared to controls. The –log10(q-value) *Y*-axis threshold was set at 2 and denoted as a horizontal, grey dashed line. A q-value is synonymous with the false discovery rate. The % differential methylation *X*-axis threshold was set at −25 and 25 and is denoted as a vertical, grey dashed line. Hypermethylated CpG sites are denoted in green and hypomethylated CpG sites are denoted in red.

The methylation of CpG sites in the DNA of WBCs isolated from 4-month-old PI heifer calves was compared to sites from a group of age-matched control calves. Of the bisulfite-converted sequence reads, an average of 32.4% were aligned to a single location within the reference genome, and 51.4% were aligned at more than one location within the reference genome. The average rate of bisulfite conversion for all samples was 99.1%, and the average CpG coverage was 9X. Mean quality Phred scores were above 30 at all base positions, indicating high sequencing accuracy. Using methylKit, CpG sites with coverage in the epigenome of all control and PI samples were selected and stored for comparison. The clustering of CpG methylation demonstrated overall similarity between control and PI heifers (Figure 1A). Pearson correlation coefficients for all samples were greater than or equal to 0.95. A total of 8367 differentially methylated CpG sites (DMSs) were identified throughout the PI epigenomes when compared to control epigenomes (Figure 1B). Of the identified DMSs, 5349 (64%) sites were hypomethylated and 3018 (36%) sites were hypermethylated. Sites identified as significant had differential methylation (meth.diff) of greater than 25% and a false discovery rate of less than 0.01 (Figure 1C). When the genome was analyzed by CpG islands, 317 differentially methylated regions (DMRs) were identified in PI cattle compared to controls. Similarly, when the epigenomic data were compared based on promoter regions, 182 DMRs were identified in PI cattle.

**Figure 2:**
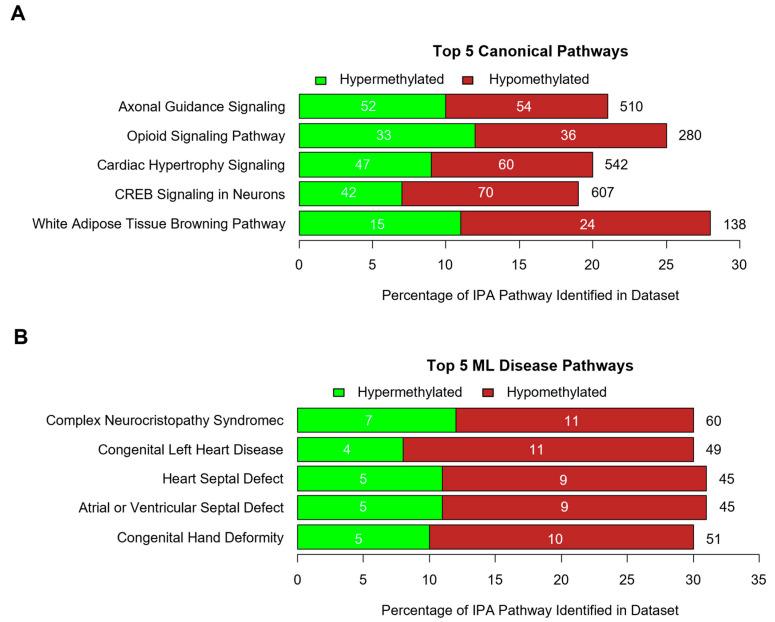
IPA-predicted impacts on PIs through the utilization of differentially methylated CpG sites. (**A**,**B**) Top 5 canonical pathways and ML disease pathways as determined by Ingenuity Pathway Analysis. Green denotes hypermethylation while red denotes hypomethylation. The total number of genes included in the IPA pathway is denoted to the right of each stacked bar. The numbers of hypo- or hypermethylated genes identified in the dataset are denoted within each stacked bar.

Data collected from the differential analysis of the methylome of PI and control heifers were analyzed through IPA software. The IPA analysis summarized the most significant findings within the dataset and predicted the most affected molecules, pathways, and disorders. By comparing the number of genes identified within a dataset to the number of total genes in a pathway (defined ‘overlap’), IPA identified pathways that are likely to be altered in PI heifers. Canonical pathways in the IPA database are well-characterized and human-curated. Machine learning (ML) was utilized to generate pathways of phenotypic disease and their associated genes within the IPA database. It was predicted through IPA that several canonical pathways and ML disease pathways would be affected by methylation of the genome (Figure S1 and Figure S2). The top five canonical pathways identified include axonal guidance signaling (106/510), opioid signaling (69/280), cardiac hypertrophy signaling [enhanced] (107/542), CREB signaling in neurons (112/607), and white adipose tissue browning (39/138; Figure 2A).

The top ML disease pathways include complex neurocristopathy syndrome (18/60), congenital left heart disease (15/49), heart septal defect (14/45), atrial or ventricular septal defect (14/45), and congenital hand deformity (15/51; Figure 2B). Moreover, IPA demonstrated that a majority of the CpG DMSs identified were located on genes associated with neurotransmitters and nervous system signaling, organismal growth and development, cardiovascular signaling, cellular growth, proliferation, and development, and cancer, in order from the most to least significant (Figure S3). Genes with a strong relationship to pathologies identified in PI cattle were also grouped manually (Table 2).

### 3.3. Complete Blood Counts

**Figure 3:**
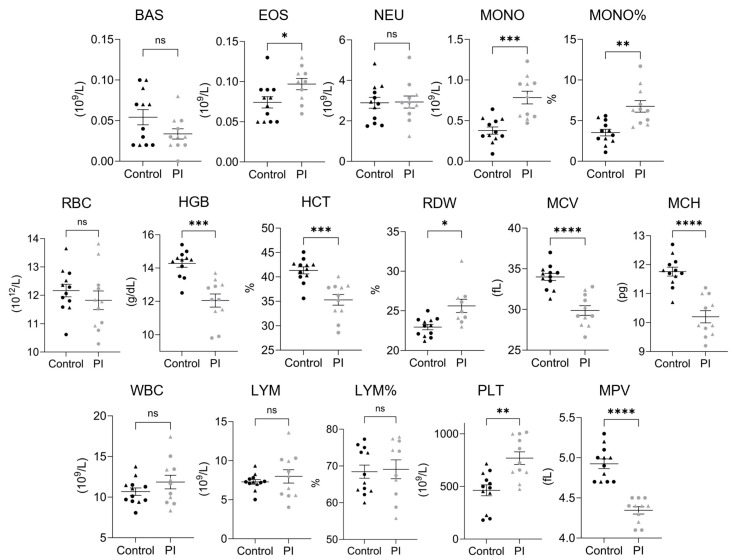
PI cattle demonstrate elevated monocytes, microcytic anemia, and abnormal platelets. Quantification of the average complete blood count values of control and persistently infected (PI) cattle. From left to right: absolute basophil count, absolute eosinophil count, absolute neutrophil count, absolute monocyte count, monocyte percentage, red blood cell count, hemoglobin, hematocrit, red cell distribution width, mean corpuscular volume, mean corpuscular hemoglobin, white blood cell count, absolute lymphocyte count, lymphocyte percentage, platelet count, and mean platelet volume. Outliers were identified using the ROUT method and were excluded from calculations of the mean and significance. Two outliers were identified in the PI group for RDW% (34 and 34.1, one of which was derived from a PI submitted for RRBS), one of which was utilized for RRBS (34.1). A single outlier within the PI group was identified for LYM% and EOS count, at 38.6 and 0.28, respectively. The 5 control and 5 PI heifers utilized for RRBS are denoted as triangles, samples not used for RRBS are denoted as circles. Data are displayed as the mean ± standard error of the mean (SEM). Asterisks denote significance between groups, * *p* < 0.05; ** *p* < 0.01; *** *p* < 0.001; **** *p* < 0.0001; ‘ns’ *p* > 0.05.

The whole blood collected from 4-month-old PI and control heifers was subjected to CBC analysis (Zoetis, Inc., Kalamazoo, MI, USA; Figure 3 and Table 3). The absolute basophil count (BAS), absolute neutrophil count (NEU), and RBCs were not different between PI and control heifers. The absolute eosinophil count (EOS) was found to be increased in PI heifers (0.07 ± 0.01 vs. 0.1 ± 0.01 × 10^9^/L; *p* < 0.05). However, the average absolute monocyte count (MONO) observed in PI heifers was approximately 2-fold higher than the average observed in control heifers (0.38 ± 0.04 vs. 0.78 ± 0.08 × 10^9^/L; *p* < 0.001). Similarly, the average percentage of monocytes (MONO%) was significantly higher in PI heifers compared to controls (3.53 ± 0.41% vs. 6.8 ± 0.71%; *p* < 0.01). Hemoglobin (HGB; 14.28 ± 0.23 vs. 12.05 ± 0.39 g/dL; *p* < 0.001) and hematocrit (HCT; 41.33 ± 0.72% vs. 35.29 ± 1.1%; *p* < 0.001) were significantly lower in PI heifers, while RBC distribution width (RDW; 22.96 ± 0.34% vs. 27.15 ± 1.23%; *p* < 0.05) was greater. Mean corpuscular volume (MCV; 33.99 ± 0.44 vs. 29.86 ± 0.6 fL; *p* < 0.0001) and mean corpuscular hemoglobin (MCH; 11.76 ± 0.15 vs. 10.2 ± 0.21 pg; *p* < 0.0001) were significantly decreased in PI heifers compared to controls, demonstrating microcytic anemia. The WBC count, absolute lymphocytes (LYM), and percent lymphocytes (LYM%) were not different between PI and control heifers. An increased platelet count (PLT; 462.8 ± 51.5 vs. 768.2 ± 60.5 × 10^9^/L; *p* < 0.01) was found in PI heifers, while the mean platelet volume was decreased (MPV; 4.93 ± 0.06 vs. 4.35 ± 0.05 fL; *p* < 0.0001).

### 3.4. Flow Cytometry

**Figure 4:**
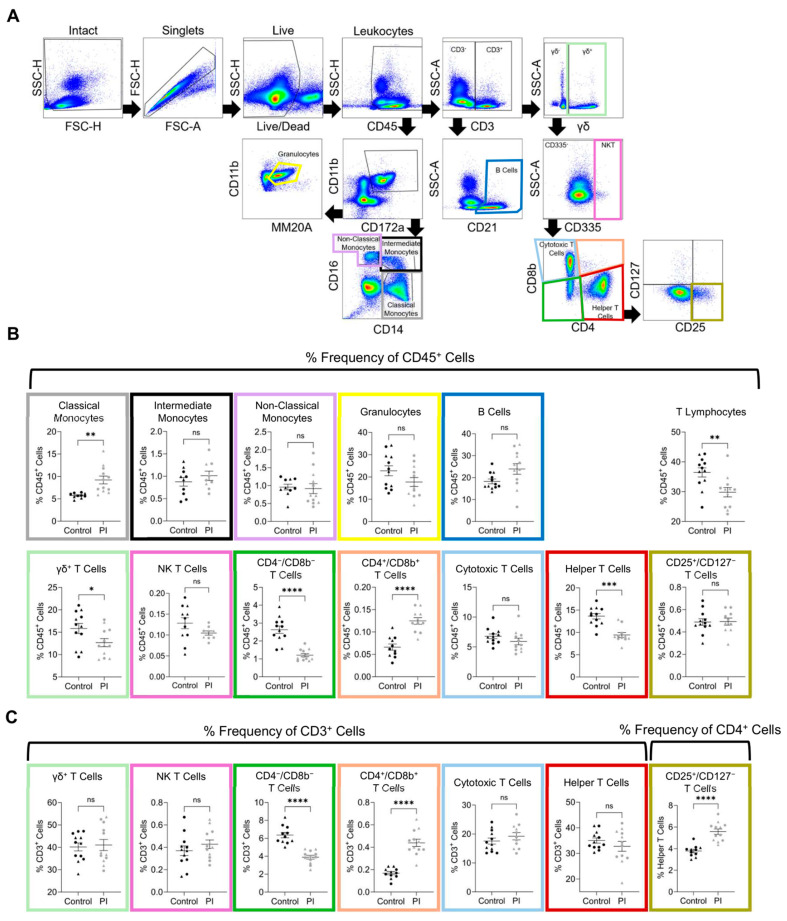
Decreased total T cells and increased subpopulations in PI heifers. (**A**) Gating strategy for the identification of T cells, B cells, monocytes, and granulocytes. Natural killer T cells are denoted as ‘NK’ cells. (**B**) Quantification of cell population frequency in relation to the CD45^+^ parent population. (**C**) Quantification of cell population frequency in relation to the CD3^+^ cell parent population. Outliers were identified using the ROUT method and removed prior to the calculation of the mean and significance. The colored boxes around each quantification of frequency corresponds to the color outlining the matching cell population on the gating strategy. Triangles denote samples from heifers utilized for RRBS analysis, circles denote samples not used for RRBS. The following outliers were identified in comparisons of CD45 frequency: classical monocytes (2.13 in the control group and 8.59 in the PI group), intermediate monocytes (2.59 and 2.76 in the control group and 2.65, 2.74, and 3.37 in the PI group), non-classical monocytes (0.17 and 2.95 in the control group), NK T cells (0.31 in the control and 0.2, 0.23, and 0.24 in the PI group), CD4^+^/CD8b^+^ T cells (0.17 in the control and 0.24 in the PI group), cytotoxic T cells (15.7 in the PI group), helper T cells (18.4 in the PI group), and CD25^+^/CD127^−^ T cells (0.84 and 1.1 in the PI group). The following outliers were identified in comparisons of CD3 frequency: CD4^−^/CD8b^−^ T cells (10.4 in the control group), CD4^+^/CD8b^+^ T cells (0.42 in the control group), and cytotoxic T cells (44 in the PI group). An outlier was also found in the comparison of CD25^+^/CD127^−^ T cells in the context of helper T cell frequency (1.93 in the control group). Italicized outliers belong to heifers utilized for RRBS. Data are displayed as the mean ± standard error of the mean (SEM). Asterisks denote significance between groups, * *p* < 0.05; ** *p* < 0.01; *** *p* < 0.001; **** *p* < 0.0001. Non-significance is denoted by ‘ns’.

To examine the effect of BVDV PI upon the postnatal immune system, flow cytometry analysis was performed on WBCs isolated from whole blood. A 20-fluorophore panel (Zoetis Inc., Kalamazoo, MI, USA) and a gating strategy were developed to identify bovine immune cell types (Figure 4A). The entire sample was first gated to remove debris, doublet cells, and dead cells. Live cells were gated based on staining for CD45, a pan-leukocyte marker. All flow cytometric comparisons were made based on the frequency of the population in relation to the CD45^+^ population unless otherwise stated (Figure 4B and Table 4).

Hematopoietic cells (CD45^+^) were selected, and CD11b and CD172a expression levels were used to define myeloid cells. Monocyte populations were identified according to CD14 and CD16 expression. Classical monocytes (CD45^+^/CD11b^+^/CD172a^+^/CD14^+^/CD16^−^) were increased in PI heifers (5.67% ± 0.43 vs. 9.21% ± 0.86; *p* < 0.01). Intermediate monocytes (CD45^+^/CD11b^+^/CD172a^+^/CD14^+^/CD16^+^) and non-classical monocytes (CD45^+^/CD11b^+^/CD172a^+^/CD14^−^/CD16^+^) were not different between the control and PI groups. Granulocytes were identified within the myeloid population by gating for MM20A expression but were not different between groups.

To identify B cells (CD45^+^/CD3^−^/CD21^+^), CD45^+^/CD3^−^ cells were gated based on CD21 expression. A significant increase in B cell percentage was discovered in PI heifers (18.28% ± 1.09 vs. 23.95% ± 2.44; *p* < 0.05). Cells expressing CD3 were then gated based on expression of the gamma delta (γδ) chain. Comparison of γδ T cells (CD45^+^/CD3^+^/γδ^+^) revealed a significantly lower percentage of γδ^+^ T cells in PI heifers (15.85% ± 1.13 vs. 12.67% ± 0.9; *p* < 0.05). Cells expressing CD45 and CD3, but not the γδ chain, were gated based on expression of CD335. Natural killer T (NKT) cells were not different between groups. Cells not expressing CD335 were further gated according to CD4 and CD8b expression to define cytotoxic T cells (CD45^+^/CD3^+^/γδ^−^/CD335^−^/CD4^−^/CD8b^+^), helper T cells (CD45^+^/CD3^+^/γδ^−^/CD335^−^/CD4^+^/CD8b^−^), and double-positive T cells (CD45^+^/CD3^+^/γδ^−^/CD335^−^/CD4^+^/CD8b^+^). A comparison of population frequency in relation to the parent population of CD45^+^ cells revealed no differences in cytotoxic T cells between groups. Helper T cells were significantly decreased in PI heifers (13.6% ± 0.61 vs. 10.18% ± 0.90; *p* < 0.01). Double-positive T cells were increased in PI heifers (0.07% ± 0.01 vs. 0.13% ± 0.01; *p* < 0.01), while cells negative for CD4 and CD8b were decreased in PI heifers (2.63% ± 0.2 vs. 1.21% ± 0.09; *p* < 0.0001). Helper T cells were gated for CD25 and CD127 expression, but no difference was identified between groups with regard to CD25^+^/CD127^−^ T cells. Total T lymphocytes (the summation of γδ^+^ T cells, NK T cells, CD4^+^/CD8b^−^ T cells, cytotoxic T cells, and helper T cells) were lower in PI heifers than in controls (36.48% ± 1.53 vs. 29.84% ± 1.57; *p* < 0.01).

**Figure 5:**
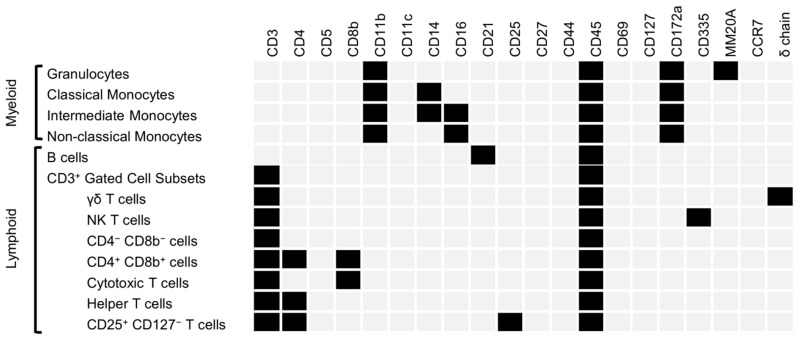
Molecular markers for cellular populations in flow cytometry. Rows indicate cellular populations. Columns indicate molecular markers. Light grey indicates that a molecular marker was not present in the population. Black shaded boxes indicate positivity for a molecular marker within a given cellular population.

Subpopulations of T cells were also compared based on frequency relative to their parent CD3^+^ cell population (Figure 4C and Table 4). Neither γδ^+^ T cells nor NK T cells within the CD3^+^ gate were different between groups. Cells positive for CD4 and CD8b were significantly increased in PI cattle within the CD3^+^ gate (6.7% ± 0.43 vs. 3.9% ± 0.21; *p* < 0.0001). Cells negative for CD4 and CD8b were significantly decreased in PI heifers (6.7% ± 0.43 vs. 3.9% ± 0.21; *p* < 0.0001). Cytotoxic T cells and helper T cells were not different between groups. However, a significant increase was identified in PI cattle when the frequency of CD25^+^/CD127^−^ cells in relation to helper T cells was compared to controls (3.63% ± 0.21 vs. 5.58% ± 0.25; *p* < 0.01). The cell population markers and results have been summarized in Figure 5.

## 4. Discussion

The cattle industry experiences billions of USD in profit loss each year due to BVDV infections [9,58]. The cost of a BVDV outbreak ranges from USD 20 to USD 103 per head, which culminates in an estimated USD 1.5 to USD 2.5 billion of loss each year [59]. Current disease mitigation strategies focus on the elimination of the main reservoir of the virus, PI calves, as they are responsible for infecting 70–100% of cattle they encounter [60]. Once PIs have been eliminated, BVDV prevention strategies depend on herd vaccination. However, due to the high antigenic diversity of BVDV, even herds that are routinely vaccinated can experience acute transient infections or fetal infections that produce PI calves [24]. Routine testing is often performed in conjunction with vaccination, identifying any transient infections occurring in the herd. To identify a PI calf, testing must be performed twice. The cost of routine testing and vaccination is significantly lower than the costs incurred by an outbreak of BVDV [59]; however, the period between initial testing and secondary testing for PI confirmation allows for additional infections and profit loss to occur. Investigation and increased understanding of potential biomarkers may provide a faster identification time for PI calves and further mitigate profit loss for producers.

### 4.1. Relationships Between PI CBCs, Flow Cytometry, and DNA Methylation

An increase in monocytes in PI cattle has been previously documented and is likely a result of chronic, ongoing infection [61]. The finding of elevated monocytes in PI calves was reinforced by flow cytometry analysis, which identified the specific population of elevated classical monocytes. It has been noted that monocytes infected with ncp BVDV1b upregulate proteins associated with cell survival [40]. It is possible that hypomethylation within *STAT6* and *IRF7* (Table 2), which is associated with the potential for increased expression, contributes to increased monocyte development and the subsequent elevation of monocytes in PI calves [41]. The increase in eosinophils seen in PI cattle is likely due to an increased parasite load, although this remains unconfirmed.

The PI calves in this study were also found to be anemic, demonstrating decreased hemoglobin, hematocrit, mean corpuscular volume, and mean corpuscular hemoglobin, as well as increased red cell distribution width. Extramedullary hematopoiesis (EMH), a condition in which the production of blood cells occurs outside of the bone marrow, has previously been observed in PI cattle [21,32]. In fact, PI fetuses experience osteopetrosis, which may be a driving factor in the observed EMH, anemia, and platelet disturbances [19,20]. While it is uncommon to see increased platelets in BVDV-infected cattle, it is likely that the circulating platelets are smaller in size due to increased age [62,63]. Moreover, BVDV infects megakaryocytes, which are responsible for platelet production [64,65].

Flow cytometry revealed that PI calves have elevated B cell levels. This is likely explained by the fact that the PI calves have been exposed to more foreign antigens compared to controls, for example, BVDV. The PI calves were also found to have decreased percentages of total T lymphocytes, γδ^+^ T cells, CD4^−^/CD8b^−^ T cells, and CD4^+^ helper T cells. Acute cp BVDV infections have been shown to decrease T cells [66,67], but PI calves are not often associated with diminished T cells [68]. In accordance with publications demonstrating decreased transcription of the T cell genes CD4, CD8a, and CD8b [15], several genes associated with T cell development were found to contain hypermethylation. These genes include *GATA3*, *NOTCH1*, *CD3*, *CD8a*, and *ZAP70*, as well as *BCL11b*, and potentially contribute to the observed decreases in T cell subpopulations (Table 2). Hypermethylation, which is associated with the decreased expression of genes necessary for T cell development, may play a role in the suppression of T cell subpopulations in PI calves, as identified by flow cytometry.

Interestingly, PI calves were found to have increased percentages of CD4^+^/CD8b^+^ T cells and CD25^+^/127^−^ T cells. Cells positive for both CD4 and CD8b display a phenotype similar to that of memory T cells, as well as high anti-viral activity [69]. Increases in double-positive T cells have been associated with immune stimulation by vaccination or infection; a recent study demonstrated that double-positive T cells are increased in bovines following foot-and-mouth vaccination and that this increase was dependent upon vaccine potency [70]. In humans, CD25^+^/CD127^−^ T cells are defined as regulatory T cells that function to suppress the immune response. However, this subpopulation of T cells is neither anergic nor suppressive of the immune system in bovine [71]. Neither of these bovine T cell subpopulations is well-characterized, leaving much to be investigated.

### 4.2. Epigenetic Findings in PI Calves Are Supported by Previous Research

The enzymes responsible for de novo methylation are most active during the development of the embryo and fetus but have been shown to remain active into the postnatal period [45]. The genes encoding these enzymes, known as DNA methyltransferases (DNMTs), were found to contain hypomethylation in a previous study utilizing DNA from splenic tissue collected from PI fetuses at gestational day 245 [32]. At 4 months of age, we also identified hypomethylation contained within *DNMT3A* and *DNMT3B* in PI heifers (Table 2). Moreover, 797 genes were found to contain differential methylation in both the DNA isolated from fetal spleens and DNA isolated from postnatal WBCs [32]. Pathways associated with previous and current studies include the immune and cardiovascular system, as well as hematopoiesis [32].

Monocytes infected with ncp BVDV have an altered profile of protein expression [72], which may reduce their ability to stimulate T cell responses [73]. A 2010 study identified 137 proteins that were differentially expressed in monocytes infected with the ncp BVDV1b NY strain [72]. Comparing the gene names from the 137 differentially expressed proteins to the differentially methylated genes in our dataset revealed 24 genes in common. Of the twenty-four genes in common, eight genes displayed differential methylation in accordance with the differential expression observed previously. The eight genes with corroborative methylation include *SH3GL1*, *MCM4*, *IGF2R*, *ECE1*, *RANBP3*, *MAN2B1*, *PDXK*, and *ANXA2* (Table 5). For example, *MAN2B1* was found to be hypomethylated at three different CpG sites and was previously observed to be upregulated in monocytes infected with ncp BVDV1b [72].

It has been reported that PI cattle have lower amounts of macrophages and that macrophages derived from PI cattle show diminished reactivity in vitro [31,72,74]. While we were unable to identify and quantify macrophages in this study, we were able to identify genes specifically associated with macrophage function that contained differential methylation in PIs. For example, the gene encoding the F-actin binding protein *EZR* was found to contain five hypermethylated DMSs (Table 2). In vitro, macrophages lacking *EZR* demonstrated impaired phagocytosis [46]. The endosomal endonuclease encoded by *PLD4* was found to contain hypermethylation of a CpG site (Table 2). Under normal conditions, the PLD4 endonuclease acts to degrade foreign genetic material that would otherwise initiate a signaling cascade to promote pro-inflammatory macrophages [47]. Perhaps the most intriguing is the similarity between PI cattle and those with deficient PLD4. Calves with insufficient PLD4 present with severe crusting dermatitis, increased vacuolated macrophages, and anemia [48]. Similarly, PI cattle are known to develop crust-like dermatosis and macrophage vacuolization, and even the PI calves identified herein suffered from anemia [11].

Previous research also indicates that PI cattle often suffer from inflammatory disorders such as myocarditis, arthritis, non-suppurative gut inflammation, and respiratory tract inflammation [75,76,77,78,79]. Consistent with these reports, several genes associated with inflammatory processes were found to contain differential methylation. Two different mechanisms that work to generate inflammation appear to be altered by PI with BVDV: the complement cascade and the activation of inflammasomes. Components of the complement cascade found to contain hypomethylation in PI calves include *C1r*, *C2*, and *C8*. Upon initiation, the complement cascade creates by-products called anaphylatoxins, which promote acute inflammation [49]. In addition, multiple inflammasome components were found to contain hypomethylation and include *NLRP6*, *NEK7*, *GSDMD*, *CASP4*, and *PANX1* (Table 2). Pro-inflammatory cytokines are also produced as a result of inflammasome activation [49]. Because PIs are continuously circulating viral particles throughout the body, chronic activation of processes involving hypomethylated genes could result in the over-promotion of inflammation and the development of secondary inflammatory diseases.

Reports of aborted PI fetuses having an ‘enlarged, flabby heart’ and an adult PI steer with myocarditis, microscopic fibrotic lesions, and necrotic cardiomyocytes indicate the involvement of the heart in chronic BVDV infections [80,81]. Epigenetic data herein further validate these reports as IPA predicted an increase in hypertrophic response, inflammation, and altered cardiac signaling. Cardiac-associated genes, including *PPP3R2*, *CALM1*, *NFATc1*, and *NFATc2*, were found to contain differential methylation in PI heifers (Table 2). A regulatory subunit of calcineurin, encoded by *PPP3R2*, was hypomethylated in PI calves. A surplus of NFATs has been implicated in the development of vascular disease and pathological cardiac hypertrophy [55,56]. Moreover, a total of forty-seven CpG sites, three CpG islands, and a single promoter were found contained within the 31 different genes encoding for potassium channels. Of the 47 DMSs, 35 were hypomethylated and 12 were hypermethylated; all CpG islands and promoters were hypomethylated. An improper amount of potassium within the heart is known to induce arrhythmia [57,82], as potassium is critically important within the heart during cardiac repolarization.

### 4.3. Potential Confounders and Future Research

While the results of this study show similarity to previous research and the documented pathologies of PI cattle, it is important to note the caveats within this dataset. The average CBC values obtained for RBCs, hemoglobin, mean corpuscular hemoglobin, and mean platelet volume are outside of the reference ranges provided with the Element HT5 Veterinary Hematology Analyzer (Heska, Loveland, CO, USA). The ranges provided by the analyzer are similar to those published by the University of California, Davis [83]. However, these references were established using mid-lactation dairy cattle with an average age of 5 years. As the calves in this study are a mere 4 months of age, the reference ranges provided are not entirely suitable for comparison; rather, the control calves generated for this study provide a more accurate comparison.

While PI calves exhibit increased CD4^+^/CD8b^+^ T cells and decreased CD4^−^/CD8b^−^ T cells, as determined by flow cytometry analysis, it is important to note that we were unable to definitively identify true double-positive or double-negative T cell populations in peripheral blood. These populations likely include true ‘double-positive’ and ‘double-negative’ T cells but may also include other cell types.

The control and treatment groups utilized in this study were of similar, but not identical, cattle breeds. Indeed, the control group generated consisted of Hereford-Angus cross cattle, while the PI calves identified were Angus cattle. While this does have the potential to induce inappropriately identified DMSs, a comparison of the *Bos indicus* methylome to that of *Bos taurus* demonstrated only a 5% bias [84]. Both groups of cattle studied herein belong to *Bos taurus*; as both control and PI cattle contained at least half Angus lineage, the expected bias due to differences in breed would be significantly lower. Additional steps were taken during analysis to further mitigate potential bias. Only CpG sites identified in all control and PI samples were selected for comparison of methylation. Thus, any variation induced by structural variance or single-nucleotide polymorphisms associated with breed was excluded from analysis. The perinatal environment also has the potential to influence the epigenome. Rather than utilizing a difference in methylation ≥10%, as is standard in other publications [84], this study opted to increase the threshold to ≥25% to identify DMSs. The increased threshold for significance was applied to mitigate individual variation and increase the reliability of results in the face of differing environments. While steps were taken during analysis to address these potentially confounding variables, it is possible that a percentage of the genes containing differential methylation identified in this study are due to differences in the breed or environment [85].

The identification of differential methylation within a gene, while capable of influencing transcription, is not guaranteed to induce pathology. For example, the gene encoding ZAP70 was found to contain hypermethylation. Canonically, the introduction of methylation within the gene could prevent the binding of transcriptional machinery and thus decrease expression. However, not all genes are being actively transcribed all the time. Because ZAP70 is involved in T cell receptor activation [49], it is most likely that this epigenetic mark would influence the ability of the calf to react to foreign antigens. The same epigenetic mark may not induce a marked impact on the heifer in the absence of immune stimulation. Additional research is required not only to confirm and validate the identification of differential methylation within specific genes but also to evaluate the impact of all identified differential methylation upon the transcription and function of potentially impacted cells in PI cattle.

## 5. Conclusions

**Figure 6:**
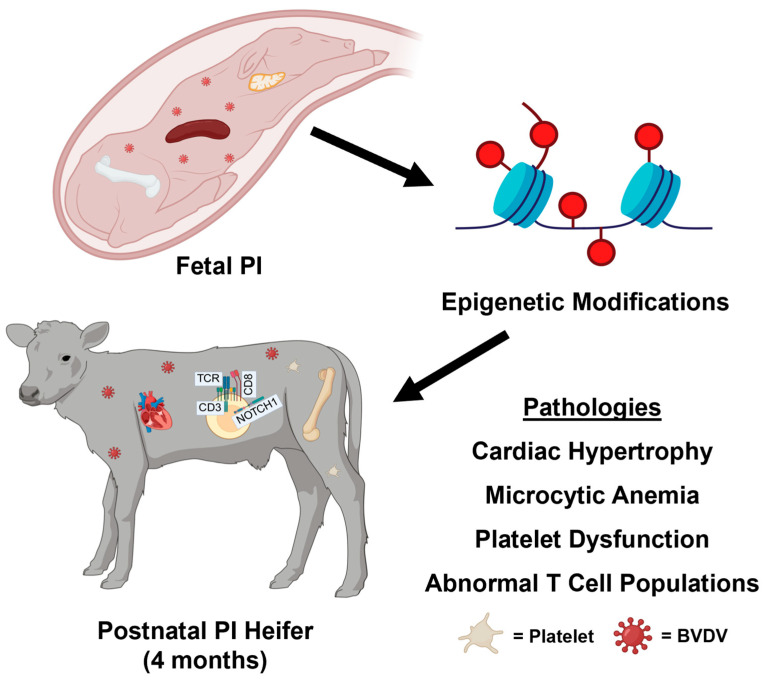
Graphical summary of results. Fetal infection with BVDV leads to epigenetic modifications that persist until the postnatal period and correspond with observed pathologies, including cardiac hypertrophy, microcytic anemia, platelet dysfunction, and abnormal T cell populations.

Research on BVDV PIs remains necessary to minimize losses in the global cattle industry. As the main reservoir of the virus, it is critical to understand how these persistent infections are established and maintained. In this study, 4-month-old PI heifers infected with a BVDV1b isolate were found to contain differential methylation of genes within the immune system, generation of blood cells, and the heart. Concurrently, complete blood count analysis at 4 months of age revealed microcytic anemia, an overabundance of small platelets, and elevated monocytes. Through flow cytometry, we determined that the PI heifers also displayed decreased populations of T lymphocytes, specifically γδ^+^, CD4^−^/CD8b^−^, and helper T cells, while also exhibiting increased percentages of CD4^+^/CD8b^+^ T cells (Figure 6). The data herein support the narrative of immunosuppression in PI heifers through the perspective of compounding epigenetic alterations through the methylation of genes. This work provides potential translatability to other vertically transmitted viruses such as hepatitis C, the Zika virus, HIV, and the Oropouche virus. With continued research and comparison, it may one day be possible to differentiate between the direct and indirect consequences of gestational viral infections.

## Figures and Tables

**Table 1:** Flow cytometry fluorophore panel. From left to right, the first column indicates the laser utilized to detect the fluorophore and associated marker. The second column lists the intended molecular antigen for identification. The third column indicates what species the antibody was intended to target. The fourth column indicates the manufacturer from whom the antibodies were obtained. The fifth column denotes the fluorophore or fluorochrome that the antibodies were stained or conjugated with. The final column on the right indicates the wavelength necessary to excite the fluorophore or fluorochrome and the wavelength at which light is emitted.

Flow Cytometry Fluorophore Panel					
Laser	Marker	Species (Clone)	Manufacturer	Fluorophore (Conjugate)	Ex/Em
405	CD11b	Anti-bovine (CC126)	BioRad	DyLight405 (conjugate)	400/420
	CD4	Anti-bovine (CC8)	BioRad	CF405 (conjugate)	408/452
	CCR7	Anti-human (3D12)	BD Horizon	BV480	436/478
	CD25	Anti-bovine (IL-A111)	BioRad	PacOrange	400/551
	CD44	Anti-mouse/human (IM7)	BioLegend	BV570	405/570
	CD127	Anti-mouse (A7R34)	BioLegend	BV605	405/605
	CD11c	Anti-human (HL3)	BD Horizon	BV650	405/645
	CD27	Anti-human (M-T271)	BD Horizon	BV711	405/711
	CD69	Anti-mouse (HI.2F3)	BioLegend	BV785	405/785
488	CD45	Anti-bovine (CC1)	BioRad	FITC	495/519
	CD14	Anti-human (M5E2)	BD	BB700	485/693
561	CD5	Anti-bovine (CC17)	BioRad	AF555 (conjugate)	553/568
	CD8b	Anti-bovine (CC58)	BioRad	PE	566/574
	CD21	Anti-bovine (CC21)	BioRad	AF568 (conjugate)	578/603
	Viability	Live/Dead	BD	FVS620	523/617
	CD335	Anti-bovine	BioRad	AF594 (conjugate)	590/618
	CD172a	Anti-bovine (CC149)	BioRad	PECy5	561/665
	MM20A	Anti-bovine	WSU	PECy7 (conjugate)	565/778
637	CD16	Anti-bovine (KD1)	BioRad	AF647	650/671
	δ chain	Anti-bovine (TCR1-N24 δ)	WSU	AF680 (conjugate)	684/707
	CD3	Anti-bovine (MM1A)	BioRad	AF750 (conjugate)	752/776

**Table 2:** Pathology-related genes containing differential methylation in PI calves. The left column contains the probable impact of the genes in the center column and the perspective methylation contained within each gene. The central column contains color-coordinated gene symbols. Red coloring indicates that the gene contained overall hypomethylation, while green coloring indicates that the gene contained overall hypermethylation. References detailing the relationship between the listed genes and the associated impact are listed on the far right.

Pathology-Related Genes Containing Differential Methylation in PI Calves		
Associated Impact	Gene Names	References
Increased monocyte survival	*STAT6, IRF7*	[40,41]
Decreased T cell development	*GATA3, NOTCH1, CD3, CD8a, ZAP70, BCL11b*	[15,42,43,44]
Improper *de novo* methylation	*DNMT3A, DNMT3B*	[32,45]
Impaired macrophage phagocytosis	*EZR*	[46]
Macrophage-induced crusting dermatitis	*PLD4*	[47,48]
Increased inflammation	*C1r, C2, C8*	[49]
Increased inflammasome activation	*NLRP6, NEK7, GSDMD, CASP4, PANX1*	[50,51,52,53,54]
Cardiac hypertrophy	*PPP3R2, CALM1, NFATc1, NFATc2*	[55,56,57]
	Hypomethylated Hypermethylated	

**Table 3:** Complete blood counts in 4-month-old heifer calves. Data in which outliers are removed are denoted using a dagger ^†^. Data are displayed as the mean ± standard error of the mean (SEM). Asterisks denote significance between groups, * *p* < 0.05; ** *p* < 0.01; *** *p* < 0.001; **** *p* < 0.0001. Reference values were derived from the Element HT5 Veterinary Hematology Analyzer (Heska, Loveland, CO, USA).

Complete Blood Counts in 4-Month-Old Heifer Calves						
	Control		PI			
Population	Average	SEM	Average	SEM	Reference	Unit
Basophil Count	0.05	±0.01	0.03	±0.01	0–0.35	10^9^/L
Eosinophil Count ^†^*	0.07	±0.01	0.10	±0.01	0–1.3	10^9^/L
Neutrophil Count	2.89	±0.27	2.93	±0.30	0.60–4.9	10^9^/L
Monocyte Count ***	0.38	±0.04	0.78	±0.08	0–1.02	10^9^/L
Monocyte Percentage **	3.53	±0.41	6.80	±0.71	0–9.5	%
Red Blood Cell Count	12.17	±0.22	11.82	±0.33	5.0–10.1	10^12^/L
Hemoglobin ***	14.28	±0.23	12.05	±0.39	8.0–14.2	g/dL
Hematocrit ***	41.33	±0.72	35.29	±1.10	23.0–42.5	%
Red Cell Distribution Width ^†^*	22.96	±0.34	25.62	±0.84	17.5–26.5	%
Mean Corpuscular Volume ****	33.99	±0.44	29.86	±0.60	37.0–55.0	fL
Mean Corpuscular Hemoglobin ****	11.76	±0.15	10.20	±0.21	12.5–18.2	pg
White Blood Cell Count	10.68	±0.46	11.86	±0.84	4.6–15.8	10^9^/L
Lymphocyte Count	7.28	±0.30	7.99	±0.86	1.5–11.8	10^9^/L
Lymphocyte Percentage ^†^	68.44	±1.79	69.10	±2.57	52.3–85.6	%
Platelet Count **	462.8	±51.5	768.2	±60.5	100–720	10^9^/L
Mean Platelet Volume ****	4.93	±0.06	4.35	±0.05	4.8–7.6	fL

**Table 4:** Flow cytometry populations in 4-month-old heifer calves. Data are displayed as the mean ± standard error of the mean (SEM). Asterisks denote significance between groups, * *p* < 0.05; ** *p* < 0.01; **** *p* < 0.0001.

Flow Cytometry in 4-Month-Old Heifer Calves					
	Control		PI		
Population	Average (%)	SEM	Average (%)	SEM	Freq. of
Classical Monocytes **	5.73	±0.18	9.21	±0.86	CD45
Intermediate Monocytes	0.88	±0.10	1.01	±0.10	CD45
Non-Classical Monocytes	0.96	±0.08	0.92	±0.14	CD45
Granulocytes	22.76	±2.24	17.75	±2.01	CD45
B Cells *	18.28	±1.09	23.95	±2.44	CD45
T Lymphocytes **	36.48	±1.53	29.84	±1.57	CD45
γδ^+^ T Cells *	15.85	±1.13	12.67	±0.09	CD45
NK T Cells	0.13	±0.01	0.10	±0.01	CD45
CD4^−^/CD8b^−^ T Cells ****	2.63	±0.2	1.21	±0.09	CD45
CD4^+^/CD8b^+^ T Cells **	0.07	±0.1	0.12	±0.1	CD45
Cytotoxic T Cells	6.78	±0.39	5.91	±0.55	CD45
Helper T Cells	13.64	±0.61	9.43	±0.54	CD45
CD25^+^/CD127^−^ T Cells	0.49	±0.03	0.49	±0.03	CD45
γδ^+^ T Cells	40.17	±1.79	41.04	±2.49	CD3
NK T Cells	0.37	±0.04	0.43	±0.04	CD3
CD4^−^/CD8b^−^ T Cells ****	6.36	±0.30	3.9	±0.21	CD3
CD4^+^/CD8b^+^ T Cells ****	0.17	±0.01	0.44	±0.03	CD3
Cytotoxic T Cells	17.55	±1.06	19.22	±1.26	CD3
Helper T Cells	35.02	±1.02	32.76	±1.93	CD3
CD25^+^/CD127^−^ T Cells **	3.79	±0.16	5.58	±0.25	CD4

**Table 5:** Differentially expressed genes in PI-derived monocytes containing differential methylation in PI WBC DNA. The leftmost column contains gene symbols for those identified to contain differential methylation in PI cattle in correspondence with those differentially expressed in PI-derived monocytes. The central column contains an indication of whether the gene was found to contain a hyper- or hypomethylated CpG site. In cases where the gene contained more than one differentially methylated CpG site, ×N indicates the number of sites identified. The differential expression of genes identified in PI-derived monocytes listed in the right column was derived from [72].

Differentially Expressed Genes Containing Differential Methylation		
Gene Symbol	Methylation	Expression
*SH3GL1*	Hyper	Decreased
*MCM4*	Hyper	Decreased
*RANBP3*	Hyper ×/Hypo ×1	Decreased
*IGF3R*	Hypo	Increased
*MAN2B1*	Hypo ×3	Increased
*PDXK*	Hypo ×3/Hyper ×1	Increased
*ANXA2*	Hypo ×2	Increased

## Data Availability

The data presented in this study are openly available in the NCBI GEO Database at https://doi.org/10.5281/zenodo.13249639, accessed on 5 May 2025, reference number GSE271244.

## Supplementary Materials

The following supporting information can be downloaded at: https://www.mdpi.com/article/10.3390/v17050708/s1, Figure S1: Ingenuity Pathway Analysis-predicted canonical pathways; green denotes hypermethylation while red denotes hypomethylation. The total number of genes included in the IPA pathway is denoted to the right of each stacked bar. The numbers of hypo- or hypermethylated genes identified in the dataset are denoted within each stacked bar. Figure S2: Ingenuity Pathway Analysis machine learning (ML) disease pathways; green denotes hypermethylation while red denotes hypomethylation. The total number of genes included in the IPA pathway is denoted to the right of each stacked bar. The numbers of hypo- or hypermethylated genes identified in the dataset are denoted within each stacked bar. Figure S3: Ingenuity Pathway Analysis pathway categories; canonical pathways predicted by IPA software are grouped into functional categories. The size of the bubble is determined by the number of differentially methylated genes identified in PI heifers in relation to the total number of genes within the IPA pathway. The bubble’s color corresponds to its z-score: orange indicates a positive score and blue denotes a negative score.
